# Primary pulmonary plasmacytoma: a case report introduction

**DOI:** 10.1186/s12957-016-0948-8

**Published:** 2016-08-04

**Authors:** Si Nie, De-Chang Peng, Hong-Han Gong, Cheng-Long Ye, Xiao Nie, Hai-Jun Li

**Affiliations:** Department of Radiology, The First Affiliated Hospital of Nanchang University, No. 17, Yongwai Zheng Street, Donghu District, Nanchang, 330006 Jiangxi Province People’s Republic of China

**Keywords:** Primary pulmonary plasmacytoma, Extramedullary plasmacytoma, CT scan

## Abstract

**Background:**

Extramedullary plasmacytoma is a rare plasma cell neoplasm within soft tissue and without bone marrow involvement or other systemic characteristics of multiple myeloma. Primary pulmonary plasmacytoma is a rare type of extramedullary plasmacytoma.

**Case presentation:**

A 48-year-old male with a tumor in the right middle ear was referred to our hospital. A routine chest X-ray was arranged and showed enlargement of the left lung hilum. His bilateral breathing sounded clear. A chest CT scan revealed a well-circumscribed mass. Pathological biopsy yielded a diagnosis of isolated pulmonary plasmacytoma.

**Conclusions:**

This is the first presentation of primary pulmonary plasmacytoma with a solitary pulmonary nodule and no lymph node involvement.

## Background

Extramedullary plasmacytoma (EMP) comprises approximately 3–5 % of all plasma cell neoplasms. Eighty percent of EMP occurs in the head and neck, and most cases involve the upper aerodigestive tract [1]. Primary pulmonary plasmacytoma (PPP) is a rare type of extramedullary plasmacytoma. In a comprehensive literature search reviewing patients with EMPs in English language literature, only 11 reports were found [2–10] (summarized in Table 1), and cases that were described include clinical therapy and prognosis. Here, we present an extremely unusual presentation as a pulmonary mass and without bone marrow involvement or other characteristics of multiple myeloma.

**Table 1:** Summary of the literature on the clinical treatment and prognosis of primary pulmonary plasmacytoma

Author, year	Age	Gender	Extension	Treatment	Prognosis
Nozomi Niitsu, 2005 [9]	71	Woman	A tumor in the right middle lobe	Chemotherapy	After completing three courses of the therapy, considerable diminution in the size
Montero C, 2009 [6]	59	Man	A tumor in the left main bronchus and enlarged subcarinal lymph nodes	Surgical and radiotherapy	Currently asymptomatic and has remained disease free during a follow-up of 10 years
Montero C, 2009 [6]	64	Man	A mass in the right upper lobe	Radiotherapy	A disease-free period of 15 years followed
Montero C, 2009 [6]	56	Man	A mass and reduced right upper lobe volume	Radiotherapeutic and adjuvant chemotherapy	Developed fever and signs of septic shock during the third cycle and died
Geetha Joseph, M.D. [2]	79	Man	A right hilar mass	Right middle lobectomy	Not mentioned
Sang-Heon Kim, 2012 [5]	26	Woman	Infiltrative lesions in both lower lung fields	Chemotherapy	Near complete radiological resolution was observed after six cycles of treatment
Z. Mohammad Taheri, 2010 [4]	60	Woman	Bilateral alveolar consolidation	Chemotherapy	After four monthly courses, the chest X-ray became normal
Shi-Ping Luh, 2007 [7]	42	Woman	Right anterior mediastinal shadow with multiple pulmonary nodular lesions.	Surgical and chemotherapy	Symptoms improved after 2 months of treatment
Takahiro Horiuchi, MD, 1998 [10]	45	Woman	Massive parenchymal infiltrate in the lower lobes	Chemotherapy	After four monthly courses, the chest X-ray became normal
Lenara Renó Arbex Coelho, 2015 [8]	53	Man	Ovoid opacity in the right hilar region	Radiotherapy	After 3 years, no suspect finding of disease recurrence/progression
James N. Wise, 2001 [3]	65	Man	A right hilar mass	A right thoracotomy with right upper lobectomy	Fifteen months postoperatively, without evidence of recurrence

## Case presentation

A 48-year-old male with a tumor in the right middle ear was referred to our hospital. A routine chest X-ray was arranged and showed enlargement of the left lung hilum. His vital signs were as follows: blood pressure 117/75 mmHg, pulse 93/min, breathing 20/min, body temperature 36.3 °C. There was no systemic or superficial lymph node enlargement, sinus area tenderness, or swollen tonsils. The trachea was in the mid-line. The patient had a barrel chest. His bilateral breathing sounds are clear. Bronchoscopy revealed no obvious abnormal findings. The patient underwent a series of evaluations such as serum calcium, urine Bence-Jones protein, and plasma electrophoresis for M protein detection. However, all of the above tests were negative. A bone marrow biopsy revealed normal patterns of cell distribution. He had an approximately 10-year history of smoking. He had a tumor in the right tympanic cavity surgically removed with a postoperative pathological diagnosis of extramedullary plasmacytoma. He had no history of tuberculosis (TB). No family members had any similar clinical manifestations nor had any died of similar diseases.

**Fig. 1:**
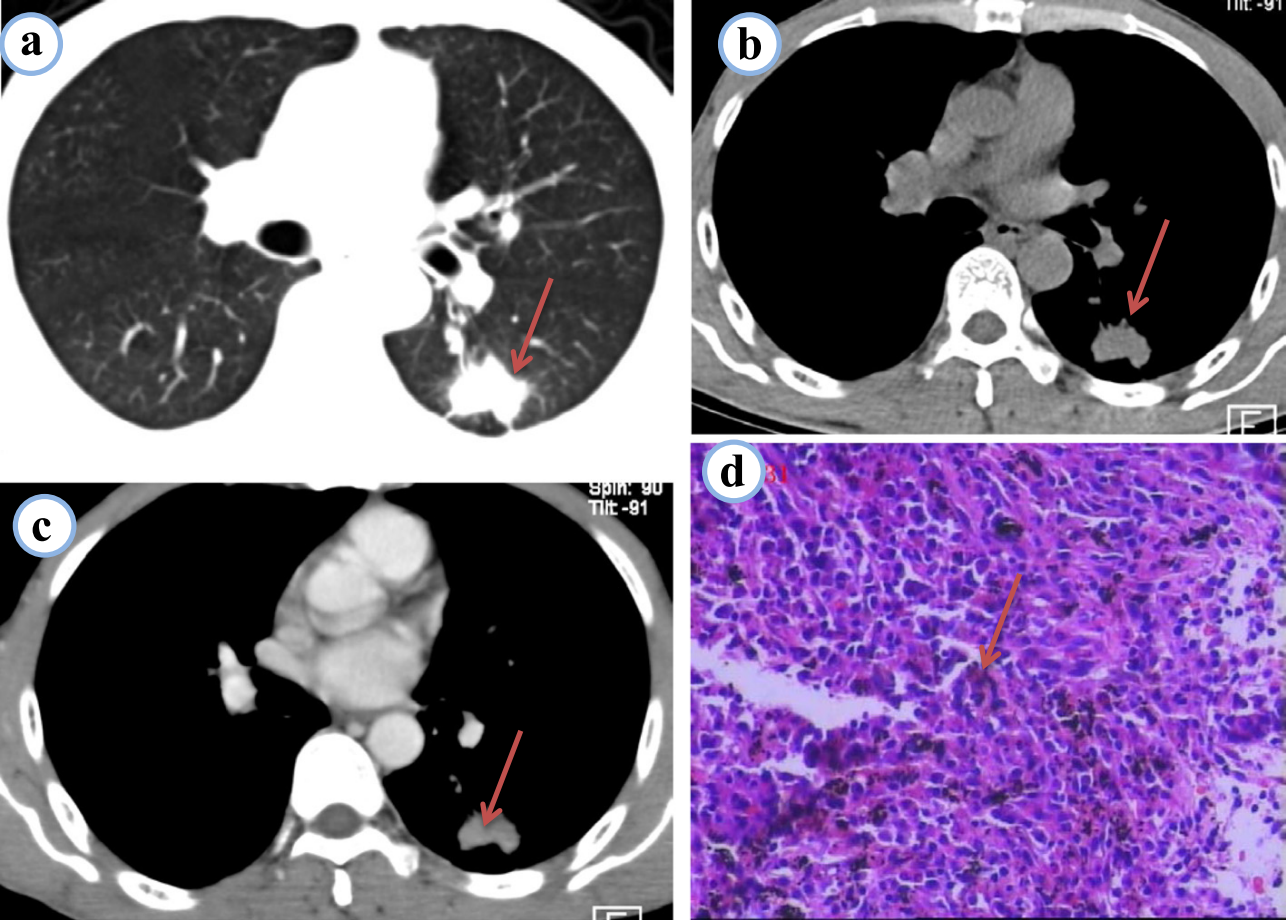
**a**~**d** Pulmonary plasmacytoma. **a** Chest CT lung window shows a well-circumscribed mass in the inferior lobe of the left lung, fine burrs in marginal lobulated, adjacent pleural retraction. **b** Chest CT mediastinal window on a plain scan shows that the nodule was homogeneous. **c** CT enhancement scanning shows that the nodule displayed moderate uniform reinforcement; **d** Microscope shows more amount of plasma cell distribution, large cells, nuclear round or ovoid, same size, often offset, chromatin spokes shaped, generally no nucleoli, nuclear fission as rare, abundant cytoplasm, many basophils (HE × 100)

A chest computed tomography (CT) scan demonstrated a well-circumscribed mass measuring 2.7 × 1.5 × 2.5 cm located in left lower lobe dorsal segment (Fig. 1a, b). The mass was homogeneous and without any area of calcification or necrosis on a CT plain scan. It was marginal, lobulated, and spiculated with adjacent pleural retraction and caused bronchiolar obstruction. Enhanced scanning revealed that the nodule displayed moderate uniform reinforcement, and tiny blood vessels could be observed (Fig. 1c). No obvious enlarged lymph nodes were found in the mediastinum. CT data resulted in a diagnosis of peripheral lung cancer. A skull, spine, and pelvis X-ray revealed no osteolytic lesions.

A CT-guided needle aspiration biopsy specimen showed that, microscopically, plasma cells were situated in the fibrous tissue, and the cells were larger than normal with Russell bodies. Small blood vessels were also revealed (Fig. 1d); immunohistochemical kappa (+++) predominate, lambda (−), CD20 (−), CD79a (−), CD138 (+++), CD38 (+++). Pathological biopsy indicated isolated pulmonary plasmacytoma.

The patient was treated with chemotherapy alone. After 6 months, the chest X-ray became normal and the patient was free of symptoms. The patient remained disease free during a follow-up of 1.5 years. Now, he is still in follow-up.

### Discussion

Plasma cell neoplasms can be classified into the following types: multiple myeloma (bone marrow and other systemic involvements), solitary myeloma (bone plasmacytoma), extramedullary (soft tissue) plasmacytoma, and plasmablastic sarcoma [11].

#### Clinical manifestations

EMP affects males three to four times more often than females, with an average age of 55. However, one third of patients with EMP are under 50 years old. The etiology of the disease is not well understood, but viral pathogenesis and chronic irritation are suggested to be contributing factors [12]. It presents with non-specific symptoms, such as chronic cough, dyspnea, wheezing, huskiness, or hemoptysis. The presentation is closely related with lesion location. Violation of the trachea can result in a series of symptoms such as breathlessness and difficulty of breathing; bronchial tumor invasion may appear as blood in phlegm or hemoptysis. If there is any involvement of the pleura, the patient may experience symptoms such as chest pain and systemic symptoms are not obvious. In this case, the patient may not exhibit symptoms for a long time, have no obvious symptoms of cough, or coughing up phlegm or symptoms such as chest pain. The clinical staging of PPP was generally based on the Wilshaw method, divided into three stages as follows: stage I: the tumor is confined to the primary site; stage II: the tumor has invaded local lymph nodes; stage III: there are obvious widespread metastases. Therefore, in this case, the clinical staging should be classified as stage I.

#### Laboratory examination

Solitary extramedullary plasmacytoma of the lung is extremely rare; pulmonary involvement with multiple myeloma is more common.

To differentiate solitary EMP from multiple myeloma, bone marrow examination is required. The patient should have lower than 5 % plasma cells with no dyscrasia and a normal skeletal survey. Unlike multiple myeloma, EMPs may not have serum M protein or Bence-Jones light chains in the urine. After treatment, the review should detect the M protein level, and testing a bone marrow is essential for ruling out multiple myeloma.

#### Histopathological characteristics

PPP microscopically showed dense plasma cells and different levels of diffuse proliferation and infiltration. Mature and relative mature nuclei are round or ovoid, of the same size, often offset, with spoke-shaped chromatin and generally no nucleoli. Nuclear fission is rare and cytoplasm is abundant. These cells are primarily basophilic and the minority as eosinophilic. The nucleus is peripheral or one side is surrounded by perinuclear halos. Irregular immature plasma cells have a low nuclear membrane thickness, scattered chromatin n spoke-shaped arrangement, and clear dual-core nucleolus. Nuclear fission is common. Cells have less cytoplasm and are basophilic or eosinophilic. Immunohistochemical staining reveals single light chain expression, namely kappa lambda (−) or (+) or kappa lambda (+/−) predominate. Tumor cells were CD20 (−), CD79a (++), CD138 (++), and CD38 part (+) [4]. These features are in accordance with the typical histopathological characteristics of PPP. The immune phenotype is highly specific for the diagnosis of plasmacytoma, especially the expression of CD138.

#### Image diagnosis and differential

The CT findings of PPP are more likely to be solitary pulmonary nodules or shadow masses, localized to the lung hilar region, with a tendency to be in the lower lobus, forming a round or round-like shape 1.5~6.0 cm in diameter, with relatively uniform density [13] occasionally empty and clear edges. PPP displaying multiple shadowed masses is rare but has been reported [14]. Some patients with diffuse bilateral lung distribution can be revealed by lung consolidation through a CT scan, which implies that tumor cells infiltrate into the lung parenchyma [2, 7, 9, 10]. In this case report, the patient’s CT revealed an isolated lung nodule and no lymph node involvement. PPP usually presents as single or multiple nodules in the lung so is often misdiagnosed as focal organizing pneumonia, tuberculosis, or lung cancer (summarized in Table 2).

**Table 2:** CT diagnosis and differential diagnosis of PPP

	PPP	Peripheral lung cancer	Tuberculoma	Nodular focal organizing pneumonia
Lobule	Superficial lobe	Deep lobules	No lobule	No lobule
Rim	Fine burr shadow	Short hard burrs	Long burrs, satellite lesions	Peripheral visible burrs
Density	Relatively uniform density, occasionally empty	Most uneven, jagged hole in the inner wall	Calcification, hollow with smooth inner wall	Uneven density, pus cavity
Enhanced CT scan	Moderate uniform reinforcement	Obvious strengthening	No or light enhancement	Uniform or delayed enhancement
Pleural	Pleural retraction	Subpleural focal fatty infiltration	Pleural thickening/calcification	Adjacent pleural thickening

In this case, the presentation was isolated lung nodules, lobulated, rim visible, fine burr shadow, and pleural retraction and was initially misdiagnosed as peripheral lung cancer. However, that condition is often characterized by deep lobules, short hard burrs, accompanied by jagged cavity, and lesions containing stiff gas bronchial wall that may be truncated, and involvement of the pleura can blur the subpleural fat gap. An enhanced CT scan showed obvious strengthening at approximately 20~60 HU.

In this case, the lesion location in left lower lobe dorsal segment was characterized by isolated soft tissue nodular shadows, differentiating it from tuberculosis. Tuberculoma often presents with calcification, more satellite lesions around the nodule, hollow with smooth inner wall, and the enhanced scan is no or light improved. There are clinical case reports of PPP lesions with calcifications [15], so calcification does not exclude the possibility of PPP. Therefore, a clinical laboratory examination is necessary for the diagnosis of tuberculosis.

In this case, it showed an isolated lung lesion with a less clear boundary, which also differentiates it from nodular focal organizing pneumonia. The edge of focal organizing pneumonia can be clear or fuzzy, its lesions have uneven density, liquefied necrotic tissue forms hollows, and it presents with peripheral visible burrs. Enhanced scanning can show lesions uniform or peripheral enhancement with adjacent pleural thickening and pus cavity.

#### Treatment and prognosis

Primary pulmonary plasmacytoma consisting of a solitary lesion is usually treated by either surgical resection alone or resection followed by radiation therapy. But the previous three cases of diffuse pulmonary infiltration and one case of multiple lung nodules, as well one case of a tumor in each lung underwent combination chemotherapy including melphalan and prednisolone [5, 9, 10]. In this case, because of the small size of the lesion, we recommended chemotherapy to patients who previously had a history of surgery and in poor health.

## Conclusions

We presented a case of PPP with an isolated lung nodule and no lymph node involvement. An isolated lung nodule showed superficial lobe, fine burr shadow, pleural retraction, and moderate uniform reinforcement; we should take into account the possibility of PPP. When the mass is small, not appropriate for operation, we can put chemotherapy as the preferred solution.

## Abbreviations

CT, computed tomography; EMP, extramedullary plasmacytoma; PPP, primary pulmonary plasmacytoma; TB, tuberculosis
